# Supplementary material for: Clinical-grade autonomous cytopathology through whole-slide edge tomography
Source: Nature. 2026 Feb 18;651(8105):472–81. doi: 10.1038/s41586-025-10094-y (PMC12979202; doi:10.1038/s41586-025-10094-y)
Supplement: Supplementary file 1 — Supplementary Figs. 1–4 and Tables 1 and 2. [file 41586_2025_10094_MOESM1_ESM.pdf]

nature portfolio

https://doi.org/10.1038/s41586-025-10094-y

Supplementary information

Clinical-grade autonomous cytopathology through whole-slide edge tomography

In the format provided by the authors and unedited

Supplementary Information

Clinical-grade autonomous cytopathology via whole-slide edge tomography

Nao Nitta[1*+], Yuko Sugiyama[2,4+], Takeaki Sugimura[1], Takahiko Ito[2], Koichi Ikebata[2], Hitoshi Abe[2], Shuhei Ishii[3], Hiroyuki Kanao[4], Nagisa Hosoya[1], Raihan Ull Islam[1], Aditya Jain[1], Meisam Hasani[1], Joseph Zonghi[1], Peter Koh[1], Yukihito Mase[1], Miki Kanematsu[1], Noureldin M. Z. Ali[1], Yoshihiko Murata[5], Ayumi Shikama[6], Yusuke Kobayashi[6], Daisuke Matsubara[5], Yukari Himeji[7], Hiroshi Nakamura[8], Akane Hashizume[8], Miyaka Umemori[9], Hiroyuki Ohsaki[9], Yingdong Luo[10], Tianben Ding[10], Fernando C. Schmitt[11], Robert Y. Osamura[1,12], Tomohiro Chiba[2,3*], & Keisuke Goda[1,10,13,14,15*]

1. *CYBO, Tokyo 135-0064, Japan*
2. *Department of Cytology, Cancer Institute Hospital of JFCR, Tokyo 135-8550, Japan*
3. *Department of Pathology, Cancer Institute Hospital of JFCR, Tokyo 135-8550, Japan*
4. *Department of Gynecology, Cancer Institute Hospital of JFCR, Tokyo 135-8550, Japan*
5. *Department of Pathology, University of Tsukuba, Ibaraki 305-8575, Japan*
6. *Department of Obstetrics and Gynecology, University of Tsukuba, Ibaraki 305-8575, Japan*
7. *Kaetsu Comprehensive Health Development Center, Niigata 957-8577, Japan*
8. *Department of Pathology, Juntendo University Urayasu Hospital, Chiba 279-0021, Japan*
9. *Department of Clinical Laboratory Technology, Juntendo University, Chiba 279-0013, Japan*
10. *Department of Chemistry, The University of Tokyo, Tokyo 113-0033, Japan*
11. *RISE-Health, Department of Pathology, Medical Faculty of Porto University, Porto 4200-319, Portugal*
12. *Department of Diagnostic Pathology, Nippon Koukan Hospital, Kanagawa 210-0852, Japan*
13. *Institute of Technological Sciences, Wuhan University, Hubei 430072, China*
14. *International Center for Synchrotron Radiation Innovation Smart, Tohoku University, Miyagi 980-8577, Japan*
15. *Department of Bioengineering, University of California, Los Angeles, California 90095, USA*

* Corresponding authors
+ Equal contribution

Table of Contents

- Supplementary Video 1
- Supplementary Video 2
- Supplementary Video 3
- Supplementary Video 4
- Supplementary Video 5
- Supplementary Video 6
- Supplementary Video 7
- Supplementary Video 8
- Supplementary Video 9
- Supplementary Video 10
- Supplementary Video 11
- Supplementary Video 12
- Supplementary Figure 1
- Supplementary Figure 2
- Supplementary Figure 3
- Supplementary Figure 4
- Supplementary Table 1
- Supplementary Table 2

Supplementary Video 1 | Keratinizing SCC (slide view). This video demonstrates the digital slide view of a keratinizing SCC. Scattered tumor cells exhibiting partial keratinization (indicated by orange coloration) are observed against a background of inflammatory cells. The viewer zooms into a representative area of interest, followed by axial navigation through Z-stack layers.

Supplementary Video 2 | Keratinizing SCC (tomographic view). This video presents the tomographic view of the same region shown in Supplementary Video 1. Spindle-shaped cytoplasmic structures of keratinizing SCC cells are clearly depicted in three dimensions. Although the sample is relatively thin (less than 10 μm), the structural details are well visualized

Supplementary Video 3 | Non-keratinizing SCC (slide view). This video presents the slide view of a non-keratinizing SCC. A densely packed cluster of tumor cells without keratinization is observed. The video includes zooming into a focal area and sequential Z-stack navigation.

Supplementary Video 4 | Non-keratinizing SCC (tomographic view). This tomographic rendering corresponds to the region displayed in Supplementary Video 3. It highlights a multilayered, sheet-like or solid structure formed by non-keratinizing SCC cells. The tumor mass demonstrates considerable cell overlapping, with a sample thickness of approximately 20 μm.

Supplementary Video 5 | HPV-associated adenocarcinoma (slide view). This video shows the slide view of an HPV-associated adenocarcinoma (usual-type endocervical adenocarcinoma). Tumor cells with a high N/C ratio and finely granular chromatin form a dense cluster, partially exhibiting tubular structures. The video navigates through a representative area of Z-stacks.

Supplementary Video 6 | HPV-associated adenocarcinoma (tomographic view). This tomographic view corresponds to the same region shown in Supplementary Video 5. The 3D reconstruction highlights the tubular architecture more clearly, demonstrating the typical morphology of HPV-associated adenocarcinoma. The sample thickness is approximately 20 μm.

Supplementary Video 7 | HPV-independent adenocarcinoma (slide view). This video depicts the slide view of an HPV-independent adenocarcinoma, classified as gastric-type mucinous adenocarcinoma. The tumor forms clusters with partially tubular architecture and features abundant mucinous cytoplasm and enlarged round nuclei.

Supplementary Video 8 | HPV-independent adenocarcinoma (tomographic view). This tomographic rendering visualizes the same region shown in Supplementary Video 7. It reveals the glandular morphology in three dimensions, with the mucin content within the cytoplasm appearing slightly yellow. The sample represents a gastric-type HPV-independent adenocarcinoma, with a thickness of approximately 15-20 μm.

Supplementary Video 9 | Fibroadenoma (slide view). This video depicts the slide view of a fibroadenoma (benign breast tumor). The ductal epithelial cells are arranged in tight sheets. Myoepithelial cells with smaller nuclei are scattered on the surface of the ductal epithelial cell sheets. Nacked nuclei are present in the background. Z-stack images clearly demonstrate that the two cell patterns of the epithelial sheets, consisting of ductal and myoepithelial cells.

Supplementary Video 10 | Fibroadenoma (tomographic view). This tomographic rendering visualizes the same region shown in Supplementary Video 9. It reveals the sheet-like or tubule-like architecture of the ductal epithelial cells, with smaller myoepithelial cells scattered on the surface. The sample represents a fibroadenoma of the breast, with a thickness of approximately 20 μm.

Supplementary Video 11 | Follicular thyroid neoplasm (slide view). This video depicts the slide view of a follicular thyroid neoplasm (follicular thyroid carcinoma). The tumor cells with mildly enlarged round nuclei form microfollicular structures containing colloids. Small follicular structures are seen scattered or overlapping.

Supplementary Video 12 | Follicular thyroid tumor (tomographic view). This tomographic rendering visualizes the same region shown in Supplementary Video 11. It clearly reveals the sphere-like microfollicular morphology in three dimensions. The sample represents a follicular thyroid neoplasm, with a thickness of approximately 15 μm.

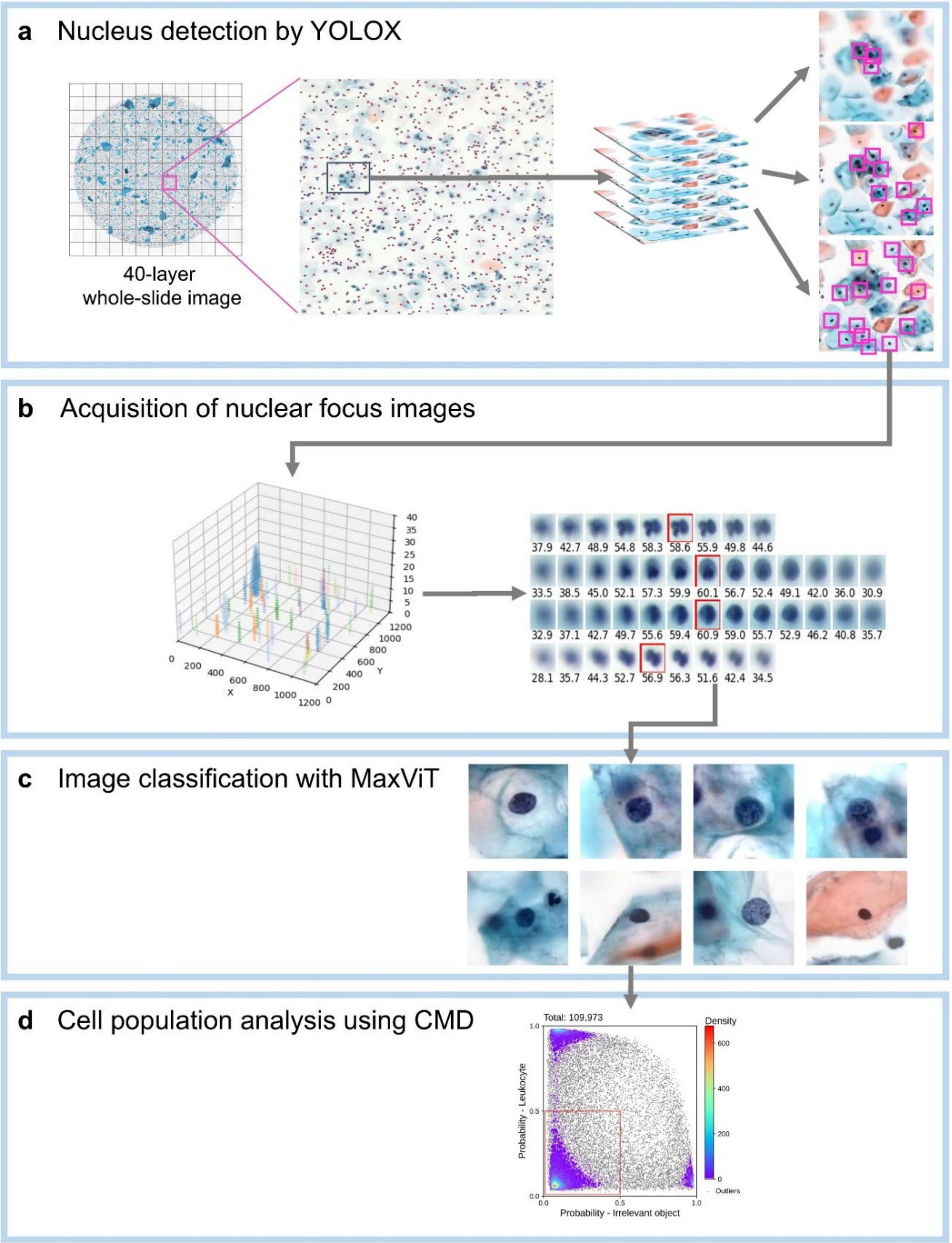

Supplementary Figure 1 | Workflow for 3D whole-slide image analysis of cervical cytology. a, Nucleus detection using YOLOX. A 2D object detection model (YOLOX) is applied to sub-sampled optical sections from the 3D Z-stack to identify nuclei. Due to the thickness of the samples, a single nucleus may appear in multiple adjacent layers and thus be detected redundantly. **b,** Acquisition of in-focus nuclear images. Redundant detections are grouped across Z-planes to define individual 3D nucleus instances (left). For each nucleus, a Z-stack of high-resolution patches is retrieved, and the most in-focus slice is selected based on a focus metric (right). **c,** Cell classification with MaxViT. A 224 × 224 pixel patch centered on the nucleus is extracted to include the full cell body. This patch is classified using a MaxViT-based vision transformer model, which outputs a CMD vector – a 10D probability vector representing the cell's morphology across multiple cytological classes. **d,** Cell population analysis using CMD. CMD vectors from all detected cells are aggregated to enable population-level analysis. This allows for visualization and identification of morphologically similar or diagnostically significant cell clusters across the whole slide.

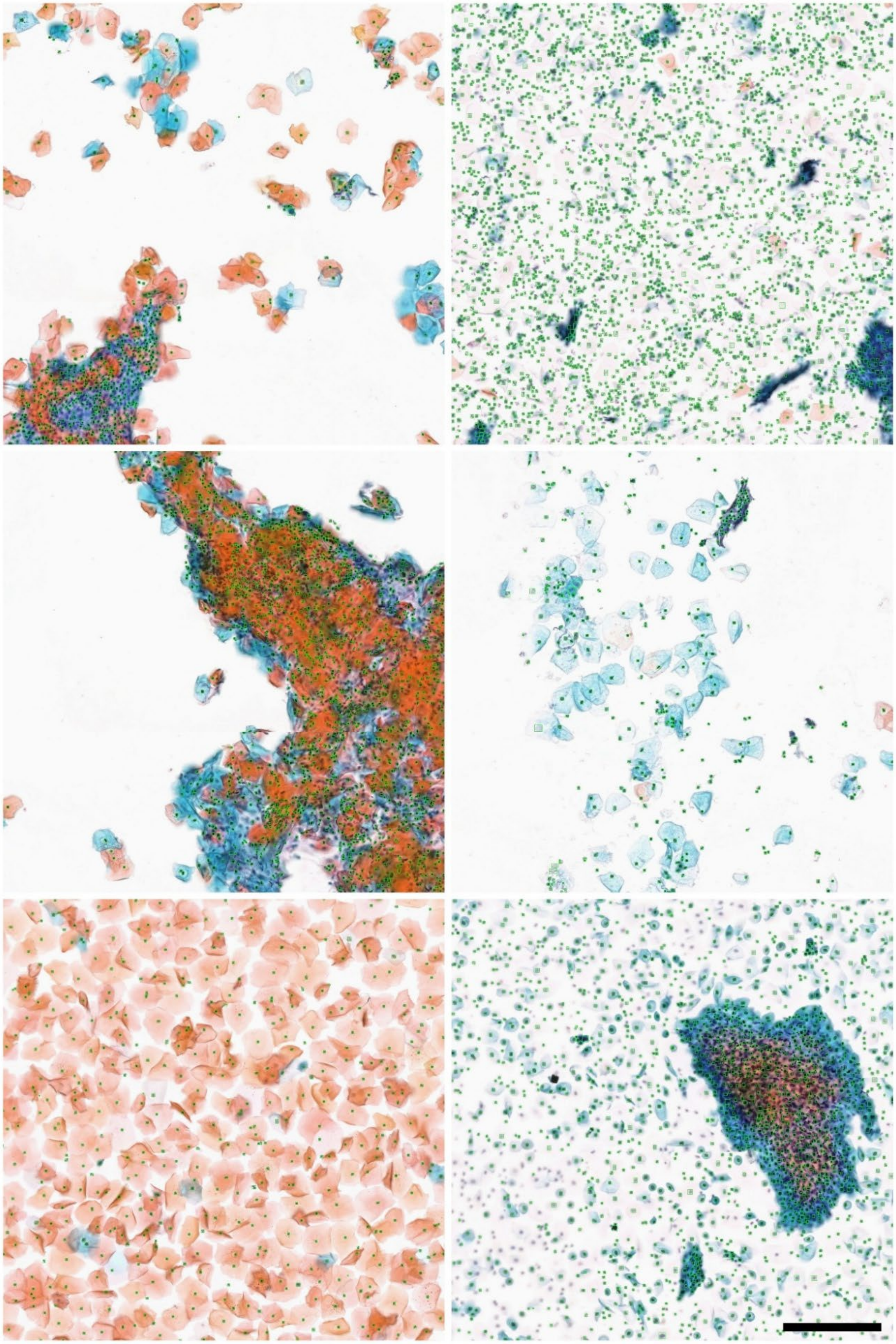

Supplementary Figure 2 | Representative training images used for YOLOX-based nucleus detection. Six representative examples from the training dataset used to develop the YOLOX object detection model (348 images in total). The selected images reflect a wide range of cytological diversity, including variations in cell type, density, and spatial arrangement, to ensure robust nucleus detection across heterogeneous sample conditions. Green bounding boxes indicate manually annotated nuclei that served as ground truth for supervised training. Scale bar: 200 µm.

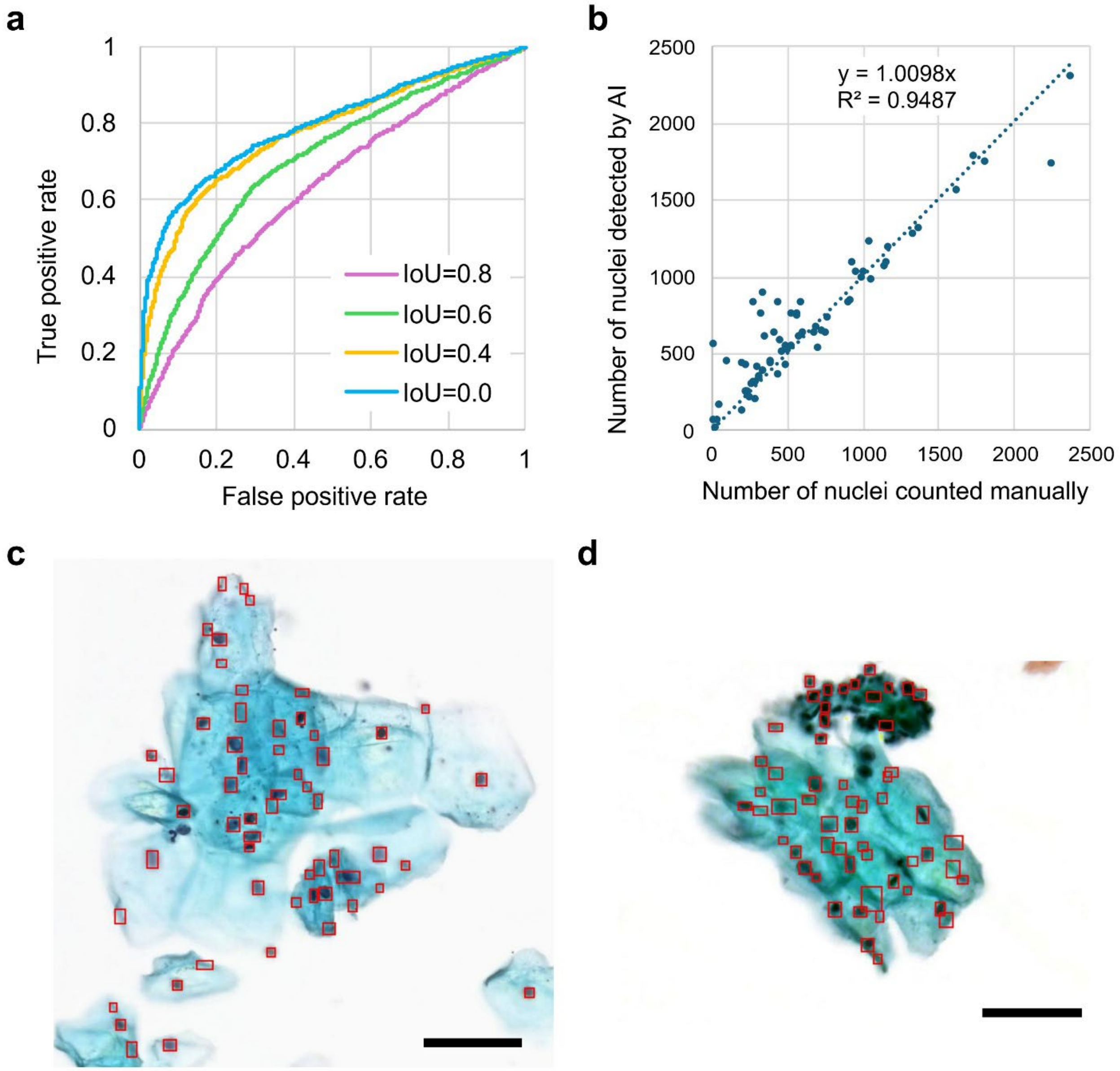

Supplementary Figure 3 | Evaluation of YOLOX-based cell nucleus detection. a, ROC curves computed at four IoU thresholds: 0.8, 0.6, 0.4, and 0.0. Model sensitivity and specificity improve as the IoU threshold is relaxed, with the highest AUC observed at IoU = 0.0. **b,** Correlation between manually counted nuclei (x-axis) and YOLOX-detected nuclei (y-axis) across the validation dataset. Each dot represents one image (n = 70). The regression line (dotted line) and coefficient of determination (R^2) indicate strong agreement. **c, d,** Representative outlier cases from panel b in which YOLOX overestimated the number of nuclei. Red bounding boxes highlight all detections, including false positives. Overestimations typically occurred at the periphery of dense cell clusters (**c**) or within background regions lacking cellular content (**d**). Scale bars: 50 μm.

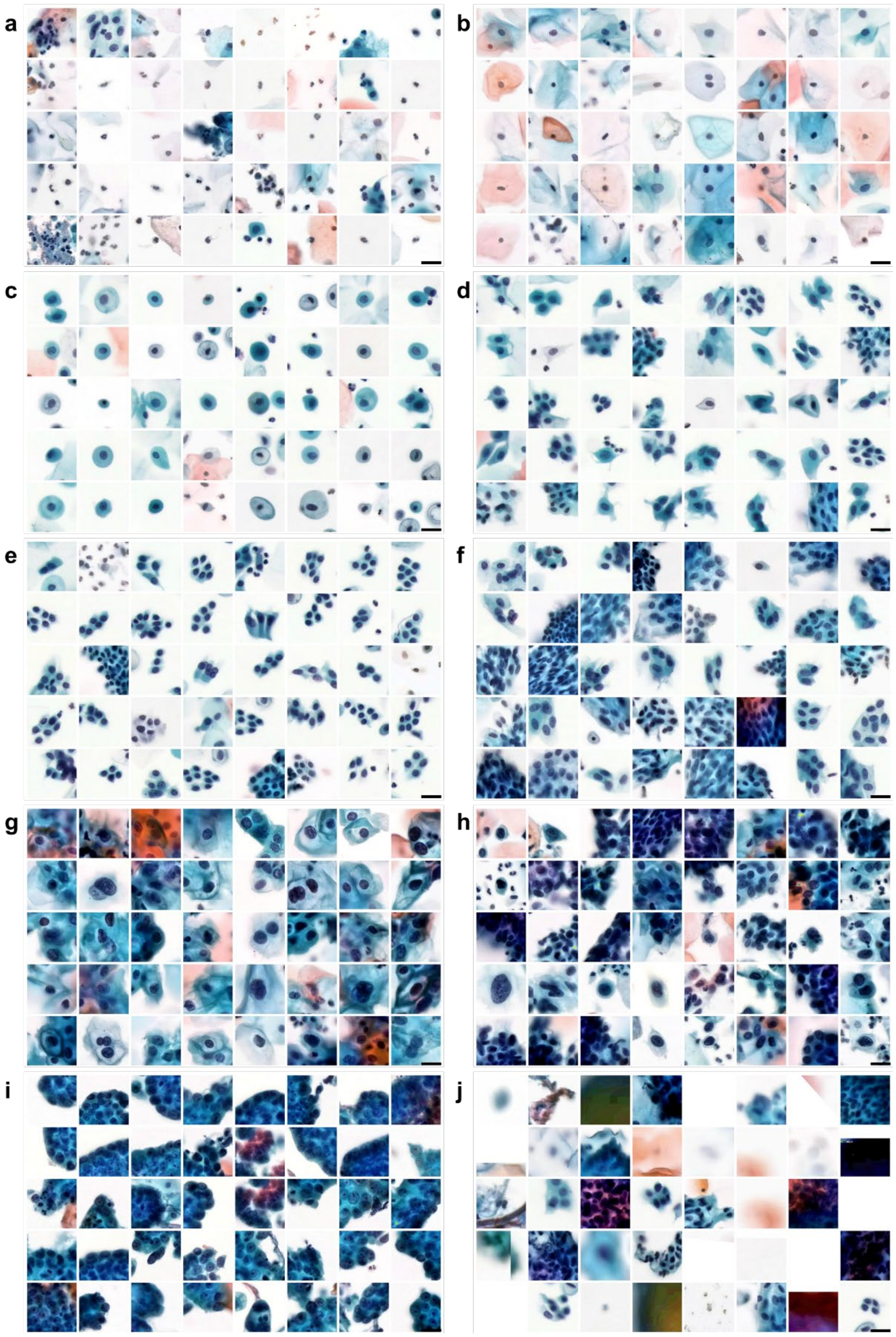

Supplementary Figure 4 | Representative training and validation images used for MaxViT-based cell classification. a, Leukocytes. **b,** Superficial/intermediate squamous cells. **c,** Parabasal cells. **d,** Squamous metaplasia cells. **e**, Glandular cells. **f**, Miscellaneous cell clusters. **g,** LSIL cells. **h,** HSIL cells. **i,** Adenocarcinoma cells. **j,** Irrelevant objects (e.g., debris, non-cellular structures, or defocused images). The complete dataset consisted of 18,219 and 5,281 leukocytes, 23,158 and 7,557 superficial/intermediate squamous cells, 4,296 and 1,243 parabasal cells, 2,056 and 487 squamous metaplasia cells, 836 and 105 glandular cells, 5,387 and 994 miscellaneous cell clusters, 1,846 and 936 LSIL cells, 1,433 and 262 HSIL cells, 912 and 420 adenocarcinoma cells, and 14,752 and 5,115 irrelevant objects for training and validation, respectively. Only a small subset of images is shown here for illustration. Scale bars: 20 μm.

Supplementary Table 1 | Sample counts by center, HPV status, and cytology category. For each center, the first row shows overall counts, followed by subtotals for HPV–, HPV+, and N/A (samples without an HPV result). Columns correspond to cytology categories, and the final column gives the total number of samples per row. Centers: Cancer Institute Hospital of JFCR (C); University of Tsukuba Hospital (T); Kaetsu Comprehensive Health Development Center (K); Juntendo University Urayasu Hospital (J). Cytology categories: NILM, negative for intraepithelial lesion or malignancy; ASC-US, atypical squamous cells of undetermined significance; LSIL, low-grade squamous intraepithelial lesion; ASC-H, atypical squamous cells, cannot exclude HSIL; HSIL, high-grade squamous intraepithelial lesion.

Center	HPV	NILM	ASC-US	LSIL	ASC-H	HSIL	SCC	Total
Cancer Institute Hospital of JFCR (C)		169	72	42	9	24	2	318
	-	84	45	5	3	2	0	139
	+	57	26	30	3	9	2	127
	N/A	28	1	7	3	13	0	52
University of Tsukuba Hospital (T)		55	31	54	17	54	11	222
	-	6	9	8	2	3	0	28
	+	10	13	36	9	39	0	107
	N/A	39	9	10	6	12	11	87
Kaetsu Comprehensive Health Development Center (K)		85	211	69	1	18	0	384
	-	41	113	8	0	0	0	162
	+	44	98	61	1	18	0	222
	N/A	0	0	0	0	0	0	0
Juntendo University Urayasu Hospital (J)		49	25	50	0	50	25	199
	-	3	6	1	0	2	0	12
	+	0	0	5	0	11	0	16
	N/A	46	19	44	0	37	25	171

Supplementary Table 2 | Summary statistics and within-center significance for AI-detected LSIL and HSIL counts. Counts are reported by center (C, T, K, J) and cytological diagnosis as n, median [Q1–Q3], mean ± SD, and min–max, aligned with the y-axis labels in Figures 5a and 5b ("Number of LSILs detected by AI," "Number of HSILs detected by AI"). One-sided Mann–Whitney U tests (a priori hypothesis: NILM < comparator) were performed within each center. Results are reported as Cliff's δ (effect size; positive when comparator > NILM) and Benjamini-Hochberg (BH)-adjusted q values (false discovery rates), with significance indicated as * q < 0.05, ** q < 0.01, and *** q < 0.001. For LSIL counts, NILM-LSIL and NILM-HSIL comparisons were significant at *** across all centers, and NILM–ASC-US reached at least * significance at every center. For HSIL counts, NILM-HSIL was *** at all other sites. Notably, for the HSIL metric, several non-HSIL categories (e.g., ASC-US, LSIL) still exhibited significant differences versus NILM at multiple centers despite much lower HSIL count levels than true HSIL slides, suggesting potential over-sensitivity of this readout.

Center	Cytology class	n	Number of LSILs detected by AI					Number of HSILs detected by AI				
			median [Q1-Q3]	mean ± sd	min-max	Cliff's δ	BH q value	median [Q1-Q3]	mean ± sd	min-max	Cliff's δ	BH q value
Cancer Institute Hospital of JFCR (C)	NILM	169	1 [0-4]	4.3 ± 10.6	0-72			0 [0-2]	2.3 ± 6.0	0-65		
	ASC-US	72	4 [2-9]	7.2 ± 12.0	0-96	0.412	0.00000021 ***	2 [0-4]	3.7 ± 5.2	0-28	0.292	0 ***
	LSIL	42	34 [11-82]	62.6 ± 85.1	2-437	0.855	6.20E-18 ***	3 [1-7]	4.7 ± 5.0	0-25	0.457	0.0000016 ***
	ASC-H	9	4 [3-9]	7.0 ± 7.7	1-26	0.517	0.004 **	6 [2-14]	13.9 ± 22.5	0-72	0.615	0 ***
	HSIL	24	18 [10-86]	53.5 ± 64.4	1-228	0.803	9.10E-11 ***	29 [12-69]	61.9 ± 104.3	0-517	0.828	7.40E-12 ***
	SCC	2	197 [117-276]	197.0 ± 224.9	38-356	0.970	0.007 **	340 [187-492]	340.0 ± 431.3	35-645	0.994	0.004 **
University of Tsukuba Hospital (T)	NILM	55	2 [0-4]	2.5 ± 2.7	0-10			8 [3-24]	28.5 ± 59.3	0-355		
	ASC-US	31	8 [1-15]	15.4 ± 31.7	0-177	0.476	0.00E+00 ***	19 [7-36]	38.7 ± 96.5	0-551	0.222	0.044 *
	LSIL	54	50 [21-293]	197.8 ± 299.6	0-1,224	0.895	1.60E-15 ***	19 [5-51]	41.1 ± 63.3	0-390	0.229	0.025 *
	ASC-H	17	3 [1-6]	4.2 ± 4.6	0-16	0.205	0.099	47 [24-191]	101.9 ± 103.9	5-319	0.644	5.60E-05 ***
	HSIL	54	21 [14-49]	47.8 ± 86.9	0-600	0.872	4.30E-15 ***	316 [209-928]	695.4 ± 833.0	22-3,798	0.949	3.30E-17 ***
	SCC	11	60 [17-130]	101.6 ± 114.9	0-339	0.848	6.60E-06 ***	2,004 [420-6,082]	4589.7 ± 5671.4	74-16,516	0.980	4.30E-07 ***
Kaetsu Comprehensive Health Development Center (K)	NILM	85	1 [0-3]	2.0 ± 3.0	0-14			3 [1-7]	6.0 ± 10.3	0-73		
	ASC-US	211	3 [1-5]	4.9 ± 8.6	0-92	0.381	2.60E-07 ***	7 [2-15]	33.0 ± 262.8	0-3,805	0.270	0 ***
	LSIL	69	10 [4-22]	28.3 ± 78.6	0-632	0.737	4.70E-15 ***	6 [2-20]	19.8 ± 33.3	0-184	0.312	0.001 ***
	ASC-H	1	9 [9-9]	9.0 ± 0.0	9-9	0.918	0.051	42 [42-42]	42.0 ± 0.0	42-42	0.953	0.052
	HSIL	18	7 [3-13]	11.8 ± 12.4	0-41	0.652	6.10E-06 ***	73 [33-223]	267.8 ± 540.9	11-2,321	0.958	4.20E-10 ***
	SCC	1	108 [108-108]	108.0 ± 0.0	108-108	1.000	0.047 *	1,019 [1,019-1,019]	1019.0 ± 0.0	1,019-1,019	1.000	0.052
Juntendo University Urayasu Hospital (J)	NILM	49	0 [0-1]	1.3 ± 3.3	0-21			2 [0-5]	6.9 ± 19.4	0-126		
	ASC-US	25	1 [0-2]	1.4 ± 2.0	0-9	0.245	0.031 *	4 [1-9]	7.6 ± 9.3	0-32	0.229	0.053
	LSIL	50	8 [4-20]	18.6 ± 29.2	0-157	0.849	2.10E-13 ***	5 [2-18]	14.1 ± 25.2	0-162	0.400	0 ***
	HSIL	50	7 [3-27]	51.3 ± 140.1	0-723	0.723	1.80E-10 ***	31 [16-109]	160.4 ± 454.9	3-3,018	0.854	4.30E-13 ***
	SCC	25	17 [3-35]	35.6 ± 53.7	0-179	0.768	1.10E-08 ***	183 [38-759]	1279.1 ± 3781.2	0-18,883	0.889	3.90E-10 ***
