# Supplementary material for: Clinical-grade autonomous cytopathology through whole-slide edge tomography
Source: Nature. 2026 Feb 18;651(8105):472–81. doi: 10.1038/s41586-025-10094-y (PMC12979202; doi:10.1038/s41586-025-10094-y)
Supplement: Supplementary file 2 — Reporting Summary [file 41586_2025_10094_MOESM2_ESM.pdf]

# nature portfolio

Corresponding author(s): Nao Nitta, Keisuke Goda, Tomohiro Chiba

Last updated by author(s): Dec 4, 2025

# Reporting Summary

Nature Portfolio wishes to improve the reproducibility of the work that we publish. This form provides structure for consistency and transparency in reporting. For further information on Nature Portfolio policies, see our Editorial Policies and the Editorial Policy Checklist.

## Statistics

For all statistical analyses, confirm that the following items are present in the figure legend, table legend, main text, or Methods section.

| n/a | Confirmed | |
|---|---|---|
| ☐ | ☒ | The exact sample size (*n*) for each experimental group/condition, given as a discrete number and unit of measurement |
| ☐ | ☒ | A statement on whether measurements were taken from distinct samples or whether the same sample was measured repeatedly |
| ☐ | ☒ | The statistical test(s) used AND whether they are one- or two-sided<br>*Only common tests should be described solely by name; describe more complex techniques in the Methods section.* |
| ☐ | ☒ | A description of all covariates tested |
| ☐ | ☒ | A description of any assumptions or corrections, such as tests of normality and adjustment for multiple comparisons |
| ☐ | ☒ | A full description of the statistical parameters including central tendency (e.g. means) or other basic estimates (e.g. regression coefficient) AND variation (e.g. standard deviation) or associated estimates of uncertainty (e.g. confidence intervals) |
| ☐ | ☒ | For null hypothesis testing, the test statistic (e.g. *F*, *t*, *r*) with confidence intervals, effect sizes, degrees of freedom and *P* value noted<br>*Give P values as exact values whenever suitable.* |
| ☒ | ☐ | For Bayesian analysis, information on the choice of priors and Markov chain Monte Carlo settings |
| ☒ | ☐ | For hierarchical and complex designs, identification of the appropriate level for tests and full reporting of outcomes |
| ☐ | ☒ | Estimates of effect sizes (e.g. Cohen's *d*, Pearson's *r*), indicating how they were calculated |

*Our web collection on statistics for biologists contains articles on many of the points above.*

## Software and code

Policy information about availability of computer code

| | |
|---|---|
| Data collection | Images were acquired using in-house developed 3D imaging hardware as described in the Methods section. Annotation data were collected using the open-source software CVAT (version 2.7.6). |
| Data analysis | Data analysis: Data analysis was conducted using Python (versions 3.10 and 3.12) with several open-source libraries, including NumPy (1.26.4), pandas (2.2.2), matplotlib (3.10.3 / 3.9.2), seaborn (0.13.2), scikit-learn (1.6.1), statsmodels (0.14.4), PyTorch (2.1.1), torchvision (0.16.1), albumentations (2.0.8), OpenCV (4.11.0.86), timm (1.0.15), and ONNX Runtime (1.18.0). Custom Python scripts for 3D image analysis, model training and inference, and visualization were developed for this study and are publicly available at Zenodo (DOI: 10.5281/zenodo.17808303; https://doi.org/10.5281/zenodo.17808303). |

For manuscripts utilizing custom algorithms or software that are central to the research but not yet described in published literature, software must be made available to editors and reviewers. We strongly encourage code deposition in a community repository (e.g. GitHub). See the Nature Portfolio guidelines for submitting code & software for further information.

## Data

Policy information about availability of data

All manuscripts must include a data availability statement. This statement should provide the following information, where applicable:

- Accession codes, unique identifiers, or web links for publicly available datasets
- A description of any restrictions on data availability
- For clinical datasets or third party data, please ensure that the statement adheres to our policy

Anonymized CSV files sufficient to reproduce the quantitative figures and tables are publicly available (DOI: 10.5281/zenodo.17808303). These CSVs contain derived measurements and per-slide metadata (including center, sample preparation method, cytology diagnosis, age and HPV test result where available) but do not include raw images or directly identifiable information. The cytology datasets analyzed in this study are securely maintained by CYBO to safeguard patient privacy and proprietary imaging data. Due to ethical and regulatory constraints, these datasets are not publicly available. Academic investigators with no relevant conflicts of interest may request controlled access to selected de-identified cytological features for non-commercial, research-only purposes. Requests will be reviewed by CYBO in consultation with the sample-providing centers and their institutional review boards or ethics committees, and, if approved, will require a data-use agreement that prohibits re-identification and any redistribution of the data. Requests should be directed to N.N. at nitta@cybo.co.jp, and eligible requests will receive a response within one month.

## Research involving human participants, their data, or biological material

Policy information about studies with human participants or human data. See also policy information about sex, gender (identity/presentation), and sexual orientation and race, ethnicity and racism.

| | |
|---|---|
| Reporting on sex and gender | The study analyzed cervical cytology samples from female patients only. |
| Reporting on race, ethnicity, or other socially relevant groupings | No data on race, ethnicity, or other socially relevant groupings were collected or analyzed. |
| Population characteristics | At the Cancer Institute Hospital of JFCR (center C), 770 cervical cytology samples were collected from patients undergoing cervical cancer screening between 2011 and 2019. Of these, 318 slides were included in the multicenter evaluation. From the other centers, 222 (University of Tsukuba Hospital, center T), 384 (Kaetsu Comprehensive Health Development Center, center K), and 199 (Juntendo University Urayasu Hospital, center J) slides were included for evaluation. Cytology categories and HPV test results are summarized in Supplementary Table 1. Age distributions are shown in Extended Data Figure 9b. |
| Recruitment | Participants were enrolled via an opt-out process in accordance with approved institutional protocols and public notification. No direct recruitment or interventions beyond routine care were performed. |
| Ethics oversight | The study protocol for the use of archived human cervical cytology specimens was reviewed and approved by the Medical Research Ethics Review Committee at the Cancer Institute Hospital of the Japanese Foundation for Cancer Research (IRB No. 2019-GA-1190; covering centers C and K), the Clinical Research Ethics Review Committee at the University of Tsukuba Hospital (R07-175), and the Research Ethics Committee of the Faculty of Health Science at Juntendo University (2025-016). All procedures were conducted in accordance with the Declaration of Helsinki and all relevant institutional and national guidelines and regulations. Informed consent for research use of archived cytology specimens was obtained via each institution's opt-out process under its broad research-consent framework, as approved by the corresponding ethics committees. All samples were anonymized and identifiable information was removed prior to analysis. |

Note that full information on the approval of the study protocol must also be provided in the manuscript.

# Field-specific reporting

Please select the one below that is the best fit for your research. If you are not sure, read the appropriate sections before making your selection.

☒ Life sciences ☐ Behavioural & social sciences ☐ Ecological, evolutionary & environmental sciences

For a reference copy of the document with all sections, see nature.com/documents/nr-reporting-summary-flat.pdf

# Life sciences study design

All studies must disclose on these points even when the disclosure is negative.

| | |
|---|---|
| Sample size | All sample sizes for each analysis are explicitly reported in the figure legends and Methods (including per-centre counts for the multicentre evaluation). No formal a priori statistical power calculation was performed. Instead, during initial development at centre C we gradually increased the number of test cases and found that datasets of approximately 200–300 test cases in total already yielded clinically meaningful and consistent estimates of key performance metrics (e.g. sensitivity, specificity, and area under the ROC curve). On this basis, and taking into account feasibility at each site, we pragmatically targeted roughly 200 test cases per centre in the multicentre study. In the final analyses, the AI system showed statistically significant superiority over human ASC-US-based triage for identifying HPV-positive cases, which we consider evidence that the chosen sample sizes were sufficient for the aims of this study. |

Data exclusions

Samples were excluded only when digital imaging failed (e.g., cellular content too sparse to permit reliable 3D imaging). These failures were identified before any statistical analysis; no post hoc exclusions were made.

Replication

Analyses were performed once per sample (one slide per donor; no technical repeats). For a given scanned slide, the image acquisition and AI analysis pipelines are deterministic, so repeating the experiment on the identical specimen would be expected to yield the same results and was therefore not pursued. Instead, we focused on assessing reproducibility and robustness at the level most relevant to the clinical application by (i) increasing the number of independent donor samples and validating performance on an independent multicentre cohort across four institutions, with AI performance summarised separately by centre and showing consistent trends, and (ii) examining the stability of diagnostic performance to analysis parameter choices. For example, in Extended Data Fig. 9e–f we varied the decision threshold from 0.60 to 0.99 in 0.01 increments and observed that the area under the ROC curve (AUC) remained stable over a broad threshold range (approximately 0.60–0.90). Taken together, these analyses support the reproducibility and robustness of our findings even though individual experiments were not technically replicated.

Randomization

This retrospective observational study involved no intervention and thus no participant randomization. At the primary center, assignment to training/validation/test splits for model development was randomized after excluding failed samples. For the multicenter evaluation, cases were target-accrued by cytological category (NILM, ASC-US, LSIL, ASC-H, HSIL, SCC) according to pre-specified targets and each center's availability, rather than by consecutive accrual or randomized allocation; all eligible cases meeting category targets were included. Performance was summarized per center to mitigate center-specific sampling imbalances.

Blinding

Annotators were not blinded to the diagnostic category of each slide for the purpose of cell-level annotations. Model training and inference were conducted on de-identified data, and predictions were generated without access to ground-truth labels; performance was computed after predictions were finalized.

# Reporting for specific materials, systems and methods

We require information from authors about some types of materials, experimental systems and methods used in many studies. Here, indicate whether each material, system or method listed is relevant to your study. If you are not sure if a list item applies to your research, read the appropriate section before selecting a response.

## Materials & experimental systems

| n/a | Involved in the study |
|---|---|
| ☒ | ☐ Antibodies |
| ☒ | ☐ Eukaryotic cell lines |
| ☒ | ☐ Palaeontology and archaeology |
| ☒ | ☐ Animals and other organisms |
| ☒ | ☐ Clinical data |
| ☒ | ☐ Dual use research of concern |
| ☒ | ☐ Plants |

## Methods

| n/a | Involved in the study |
|---|---|
| ☒ | ☐ ChIP-seq |
| ☒ | ☐ Flow cytometry |
| ☒ | ☐ MRI-based neuroimaging |

# Plants

Seed stocks

n/a

Novel plant genotypes

n/a

Authentication

n/a
